# The Care of Rheumatic Children

**Published:** 1913-01-18

**Authors:** 


### THE CARE OF RHEUMATIC CHILDREN.

Whatever the reason, climatic or racial or nutritional or what not, there is no doubt that acute rheumatism in children and young adults is a disease which is more prevalent in Britain than in most other countries. It is also indisputable that acute rheumatism is a frequent cause of valvular and other diseases of the heart; and that the prevention of these diseases is a problem of national importance. The leaders of the medical profession realised long ago the magnitude of the mischief that is done to the future citizens of the country; and their teaching has long been based on sound lines. This teaching has for years been accepted and acted upon by all the better sort of practitioners; but it is to be admitted with regret and shame that there are still many doctors whose remissness and neglect to take seriously rheumatic manifestations in the young leads to avoidable heart disease and premature death. Within the last few weeks the writer has had one such case under treatment, in which a fine strapping, healthy young man of eighteen has had his whole life's future clouded, his intended career closed to him, through the inexcusable ignorance or carelessness of a practitioner who failed to appreciate the fact that he was suffering from rheumatism and neglected both to take the temperature and to examine the heart. The mischief has been minimised at great cost and trouble, but it is impossible to repair it completely.

The time has come when the intelligent public may fitly be invited to help in the prevention of rheumatic heart disease; and this requires, first of all, acquaintance with the broad outlines of the facts connected with the disease. Such an outline is provided in Dr. Poynton's admirable lecture to a lay audience, printed on pp. 425-7 of this issue. He there explains one of the peculiarities of the disease: that although it is, as he believes, of infective origin (that is, due to invasion by a special microbe), yet the heart is the chief seat of the mischief in the young, whereas the joints are specially selected in adults. It thus follows that a mild sore throat with a little aching in the muscles or joints may, in a child, be the only symptoms of an attack of acute rheumatism, which if not properly treated may damage the heart for life. We need not repeat here the substance of Dr. Poynton's valuable address; but we feel constrained to lay stress on that paragraph in which he points out that so-called "growing pains" are in some cases really attacks of acute rheumatism, and need the most careful and long-continued attention. The public must really grasp the fact that growth is not painful, and that "growing pains" are in reality symptoms of some morbid condition, which may or may not be serious, but at least demands skilled advice. And if they can be induced to take an interest in the plain facts which Dr. Poynton sets before them, a beginning may soon be made in the tackling of a very serious national question.

